# Hunger in the Absence of Caloric Restriction Improves Cognition and Attenuates Alzheimer's Disease Pathology in a Mouse Model

**DOI:** 10.1371/journal.pone.0060437

**Published:** 2013-04-02

**Authors:** Emily J. Dhurandhar, David B. Allison, Thomas van Groen, Inga Kadish

**Affiliations:** 1 School of Public Health, Office of Energetics, Nutrition Obesity Research Center, University of Alabama at Birmingham, Birmingham, Alabama, United States of America; 2 Department of Cell, Developmental and Integrative Biology, Nutrition Obesity Research Center, University of Alabama at Birmingham, Birmingham, Alabama, United States of America; Massachusetts General Hospital and Harvard Medical School, United States of America

## Abstract

It has been shown that caloric restriction (CR) delays aging and possibly delays the development of Alzheimer's disease (AD). We conjecture that the mechanism may involve interoceptive cues, rather than reduced energy intake per se. We determined that hunger alone, induced by a ghrelin agonist, reduces AD pathology and improves cognition in the APP-SwDI mouse model of AD. Long-term treatment with a ghrelin agonist was sufficient to improve the performance in the water maze. The treatment also reduced levels of amyloid beta (Aβ) and inflammation (microglial activation) at 6 months of age compared to the control group, similar to the effect of CR. Thus, a hunger-inducing drug attenuates AD pathology, in the absence of CR, and the neuroendocrine aspects of hunger also prevent age-related cognitive decline.

## Introduction

Caloric restriction (CR), a reduction in food intake without malnutrition, increases longevity in many invertebrate and vertebrate species [1]. In addition, CR is associated with attenuation of Alzheimer's disease (AD) pathology in monkeys [2] and rodents [3]. Hence, CR may preserve cognitive function during aging through an as yet unknown mechanism. Elucidating this mechanism is vital to understanding the aging process and advancing our ability to manipulate these pathways for clinical utility. Much has been learned from the study of aging in worms and flies, but it is important to test the knowledge derived from these lower organisms in a mammalian species. The mouse is ideal for this purpose and is the most practical mammal for establishing whether homologous genes and processes can extend healthy life span. Many of the mutations that extend life span decrease activity of nutrient-signaling pathways, such as the Igf (insulin-like growth factor)/insulin and the mTOR (mammalian target of rapamycin) pathways, suggesting that they may induce a physiological state similar to that resulting from periods of food shortage [4], [5]. Furthermore, it has been suggested that Sirt1 (a mammalian member of the sirtuin gene family) contributes to the beneficial impact of caloric restriction, which may be mediated, in part, through mechanisms involving the regulation of cellular metabolism, and inflammatory and antioxidant responses [6]. Another mechanism through which SIRT1 may improve AD is by increasing α-secretase production and activity through activation of the α-secretase gene ADAM10. Because α-secretase is the enzyme responsible for the non-amyloidogenic cleavage of APP, upregulation would decrease Aβ levels [5]. Further, it has been shown that a high caloric intake based on saturated fat promotes AD type Beta-amyloidosis; conversely, it has been demonstrated that dietary restriction based on reduced carbohydrate intake is able to prevent it [7]. This evidence is consistent with current epidemiological studies suggesting that obesity and diabetes are associated with a >4-fold increased risk of developing AD. Finally, studies have shown controversial results when it comes to the relation between the adherence to a Mediterranean diet and/or physical activity and the incidence of AD. Many population-based studies have reported a positive association between the intake of polyunsaturated fat and cognitive performance [8], [9]. Similarly, it has been shown that diets high in cholesterol increase the chances of developing AD, and, furthermore, that ApoE alleles affect cholesterol metabolism and the risk of developing AD [10]–[12]. Epidemiological evidence has indicated that some statins decrease the risk of developing AD, and several preclinical studies of statin use and AD have been performed, thus far with mixed results (but see [13]). Our work, as well as that of others, has recently resulted in the development of experimental dietary regimens that might promote, attenuate, or even reverse features of aging and AD. The clarification of the mechanisms through which dietary restriction may beneficially influence AD neuropathology and the eventual discovery of future “mimetics” capable of anti-Beta-amyloidogenic activity will help in the development of “lifestyle therapeutic strategies” in aging, AD, and other neurodegenerative disorders.

The hormesis theory may provide insight into the mechanism of CR. It describes how agents, normally considered stressors, can be beneficial at low doses [14], [15]. This theory implies CR mimetics, which induce downstream CR signals such as hunger, even in the absence of CR, may be sufficient to improve age-related cognitive decline. Other authors have suggested the effects of CR may be caused by interoceptive cues such as hunger, rather than reduced caloric intake per se [16].

Ghrelin is a peptide produced by the stomach that induces hunger, and its administration increases food intake [17]. Interoceptive cues caused by ghrelin are highly similar to those produced by CR [18]. We therefore used the ghrelin agonist LY444711 [19] to test the hypothesis that hunger, in the absence of CR, is sufficient to prevent AD pathology and to prevent cognitive decline in a mouse model of AD. The LY compound we use is a ghrelin agonist. We chose to use the LY compound instead of ghrelin because it allowed us to control the dose each mouse was exposed to. This drug can be given orally and is able to withstand the digestive system and be absorbed into the bloodstream at doses that will produce hunger. Studying ghrelin itself would be more challenging, because oral administration is not an option and we cannot administer it intravenously over a long period of time, and it would thus be difficult to control the physiological dose through means other than administration. Using LY allowed us to ensure the animals were consistently hungry, as they would be during caloric restriction. This is crucial because our objective was to examine the impact of the sensation of hunger itself on AD, rather than ghrelin per se.

## Materials and Methods

### Ethics Statement

All animal protocols were approved by the University of Alabama at Birmingham IACUC, and approved protocols were strictly adhered to.

### Animals

36 male Tg APPSwDI (human APP with Swedish, Dutch and Iowa mutations on a C57BL/6 background) mice [20] at 7 weeks of age were acclimatized for 1 week to the dietary change, during that week they received food *ad libitum* and one pellet of 100 mg/day of chocolate. Mice were randomized to one of three weight-matched experimental feeding groups: control, hunger induced by LY444711 (LY), or calorically restricted (CR). The feeding period was initiated at 8 weeks of age and continued for 16 weeks. During week one of the feeding period, CR and LY groups received an equal amount of food as the control group. In week two (and the following weeks), LY animals received the same amount of food as the controls consumed during the preceding week, and the CR group received 80% of that amount (i.e., 20% caloric restriction). The LY group received 30 mg/kg/day of the ghrelin agonist LY444711 (Eli Lilly) (previously demonstrated to induce hunger in C57BL/6J mice[21]) in a chocolate pill, while control and CR animals received a placebo chocolate pill. The chocolate pill was used to hide the taste of the drug and to control the amount of drug each animal received. Animals were fed at the end of the day (around 4:00 p.m.) to mimic the “normal” feeding pattern. Animal weights were measured twice weekly, and, similarly, food intake was measured twice weekly. Animals were tested in the open field and in the elevated plus maze after 14 weeks and in the water maze after 15 weeks of treatment. Following the behavioral assessment, the animals were measured for fat mass using quantitative magnetic resonance (QMR) at the UAB Diabetes and Research Training Center Animal Physiology Core. In short, *in vivo* body composition (total body fat and lean tissue) of mice was determined using an EchoMRI™ 3-in-1 quantitative magnetic resonance (QMR) machine (Echo Medical Systems, Houston, TX). A system test was performed using a known fat standard prior to the measurements being taken. Mice were weighed and then placed into a clear holding tube capped with a stopper that restricted vertical movement but allowed constant airflow. The tube was inserted into the machine and the mouse scanned using the Normal Precision mode.

### Behavioral and cognitive assessment

The Open Field Test. The maze consists of an arena of 42 by 42 cm square with clear sides (20 cm high). The animal is put in the arena and observed for 4 minutes with a camera-driven tracker system, i.e., Ethovision (Noldus, The Netherlands). The arena is subdivided into two areas, the open center area and the sides (rearing and fecal pellets are recorded). The system records the position of the animal in the arena at 5 frames/second, and the data is analyzed as, e.g., time spent in each area, speed of locomotion, rearing, defecation, etc. This test measures activity and fear, i.e., time spent in the “open” center versus the “safe” sides.

The Elevated Plus Maze Test. The maze consist of four arms (31×5 cm) that are raised 40 cm above the table; two arms have 15 cm high sides of opaque material. The animal is put in the arena and observed for 4 minutes with a camera-driven tracker system, i.e., Ethovision (Noldus, The Netherlands). The arena is divided into the four arms, two open arms, two closed arms, and the center area, where the arms meet. The system records the position of the animal in the arena at 5 frames/second, and the data is analyzed as, e.g., time spent in each area, entries in areas, and speed of locomotion. The test measures anxiety in the animal, i.e., time spent in the open versus the closed, “safe” arms.

The water maze apparatus and procedure have been described in detail before (Liu et al., 2002). Briefly, we use a blue plastic pool, 120 cm in diameter, and a see-through round platform, 10 cm diameter, located 0.5 cm below the water surface. During day 1 through day 5 of the testing period, the mice are trained to find a hidden platform that is kept in a constant position throughout these 5 days. Three trials a day are run; all starting positions are equally used (in a pseudo-random order). The mice are given 60 seconds to find the platform and 10 seconds to stay on the platform. If the mouse does not find the platform, it is put on the platform. The inter-trial interval is approximately 1 minute. Learning of the task is evaluated by recording the swimming speed, latency to find the platform, path length, and percentage of trials each animal found the platform. After the end of the four trials on day 5 of the testing period, the mice are tested in a 60-second probe trial (i.e., trial 21), i.e., with no escape platform present. Mice that have learned the platform position will predominantly search in the “correct” quadrant of the pool in the probe trial.

### Immunohistochemistry

Animals were sacrificed after 16 weeks of the feeding period for immunohistochemical and ELISA analysis. Mice were anesthetized with Ketamine/Xylazine (100/10 mg/kg) and perfused with cold saline. The brains were removed and cut in half sagittally, and the right hemisphere of the brain was placed in 4% paraformaldehyde overnight, while the left hemisphere was dissected into four pieces (rostral cortex, caudal cortex, hippocampus and midbrain/brainstem) was frozen and stored at −80°C for ELISA analysis. The right half was put in 30% sucrose for cryoprotection, and 30 µm thick coronal sections were cut on a freezing-sliding microtome. One series of (1 in 6) sections was stained for Aβ with the W0-2 antibody (human Aβ_4–10_; 1∶2000; The Genetics Company, Schlieren, Switzerland), while one half of the second series was stained for Iba-1 (1∶1000; Wako, Richmond, VA), the other half of this series was stained for GFAP (1∶1000, Sigma). For Aβ staining sections were pretreated for 30 minutes in 85°C sodium citrate solution (pH = 6.5). Following incubation with the primary antibody in TBS-T overnight at room temperature, tissues were rinsed 3× and incubated with the appropriate biotinylated secondary antibody for 2 hours at room temperature. Sections were rinsed 3× and put for 2 hours with the tertiary antibody, extra Avidin-peroxidase, and, following another 3× rinse, metal-enhanced DAB staining was used for visualization. For each antibody, all sections were processed in one staining tray. All slides were air-dried, cleared in xylene, and coverslipped with DPX.

NIH Scion Image program was used to analyze the area occupied by Aβ and glial reactivity in stratum oriens of the dorsal hippocampus and in the dorsal dentate gyrus (for Aβ). Images of the appropriate brain areas were acquired with an Olympus DP70 digital camera. All images were acquired in one session to avoid changes in light levels.

### ELISA

The left hippocampus was homogenized in 0.5 ml of TBS with complete protease inhibitor plus 20 µg/ml pepstatin A (Roche Diagnostics GmbH, Germany) and 50 mM SDS and then centrifuged at 30,000 rpm for 60 minutes at 4°C. The supernatant (soluble fraction of Aβ) was transferred to a clean 1.5 ml tube and stored at –80°C. The pellets (insoluble fraction of Aβ) were homogenized with 400 µl of 5 M Guanidine HCl and incubated with agitation at room temperature for 4 hours. The Aβ levels of SDS fraction and Guanidine HCl fraction were quantified with an Aβ ELISA kit (Covance) according to the manufacturer's instructions. The ELISA analysis of the cytokines IL6 and IL10 (ELISA kit) were performed according to the manufacturer's instructions.

### Statistical Analysis

For ELISA, 2 technical replicates were tested. For immunohistochemistry, 3–4 sections of each brain were examined, and 3 equivalent sections (along the anterior-posterior axis of the hippocampus) were selected to be quantified, and the average of the three sections was calculated for each animal. Between-group ANOVA and post-hoc Tukey-Kramer HSD were used to make multiple group comparisons, and alpha was set to 0.05. All groups were examined using normal quantile plots for normality, and Levene's test was used to test for homoscedasticity.

## Results

**Figure 1:**
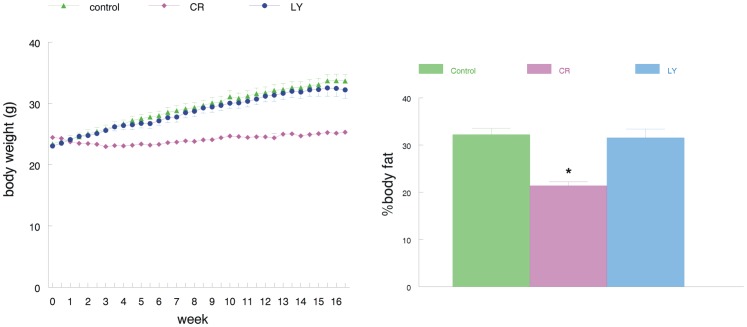
Left: Change in body weight over the treatment period. Note that the control and LY animals gain equal weight during the treatment period, but the CR animals are significantly lighter; further, the CR mice had a significantly (p<0.001) lower fat mass compared to the other two groups. Right: Bar graph showing the percent of fat of the total body weight as determined by QMR. Note the reduced fat content of the CR mice.

Both the control and LY-treatment group were fed ad libitum (i.e., the LY animals received the same amount of food as the controls consumed during the preceding week), and the calorically restricted (CR) group was fed 20% less than the control group. As expected, at the end of the study, the CR animals had a significantly lower body weight after the feeding period (p = 0.0001), while LY animals maintained their body weight similar to the controls (Figure 1). At the end of the study, all animals were analyzed for fat mass using quantitative magnetic resonance (QMR), and the data show that the CR mice had a significantly (p<0.001) lower fat mass compared to the other two groups (Figure 1).

**Figure 2:**
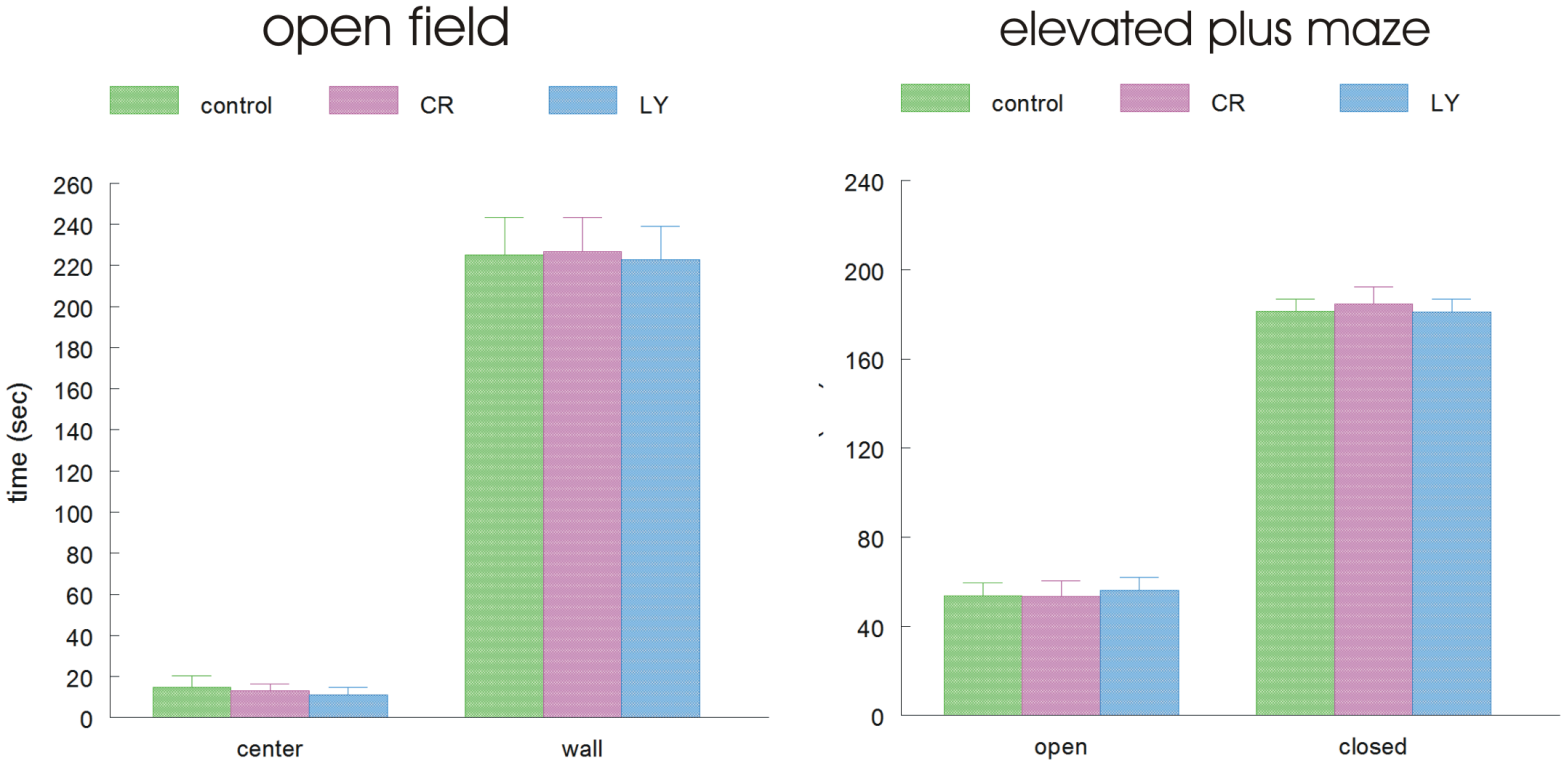
Two bar graphs showing the time spent in different parts of the open field and elevated plus maze, respectively, of the three groups of mice. No significant differences between the groups were present.

**Figure 3:**
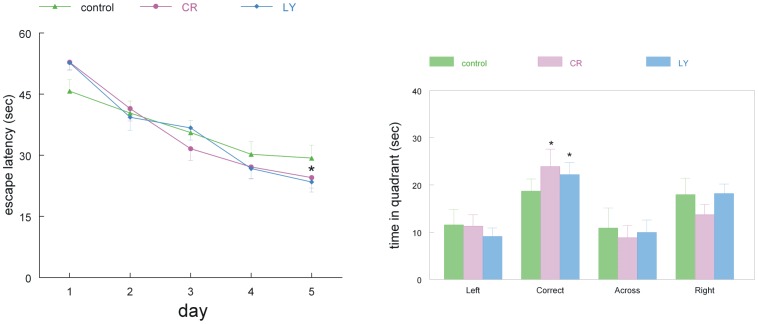
Two graphs showing the learning curves of the three groups of mice. Left: The daily mean performance in the water maze. Right: the probe trial performance of the three groups of mice. Note that the improvement in trial time was significantly more in LY compared to the control (p = 0.023) and nearly significantly more in CR compared to the control (p = 0.08). Both LY and CR groups had a significant preference for the correct quadrant in the probe trial (p<0.05).

Clinically, AD is characterized by dementia; thus, the mice were behaviorally and cognitively tested in the last two weeks of treatment, i.e., in the first week in the open field and elevated plus maze (for activity/locomotion and anxiety), followed by the water maze in the second week (cognition). There were no significant differences between groups in locomotion (time walking and speed) or time spent in the center in the open field; similarly, the time spent in the open versus closed arms of the elevated plus maze was not significantly different between groups (Figure 2). Thus, all animals had similar levels of motor activity and similar levels of anxiety. In contrast, in the cognitive testing, while all three groups learned the task, the LY group showed a significantly greater improvement in their water maze performance compared to the control group (p = 0.023). The CR group also improved but was not significantly different from the control group (p = 0.08, Figure 3). There was no significant difference in the swimming speed of the mice (control: 16.1±1.2 cm/s, CR: 15.7±1.4 cm/s, and LY: 15.6±1.0 cm/s, respectively). The results from the probe trial were not significantly different between the CR and LY groups; both groups spent significantly more time in the correct quadrant of the pool (Figure 3).

**Figure 4:**
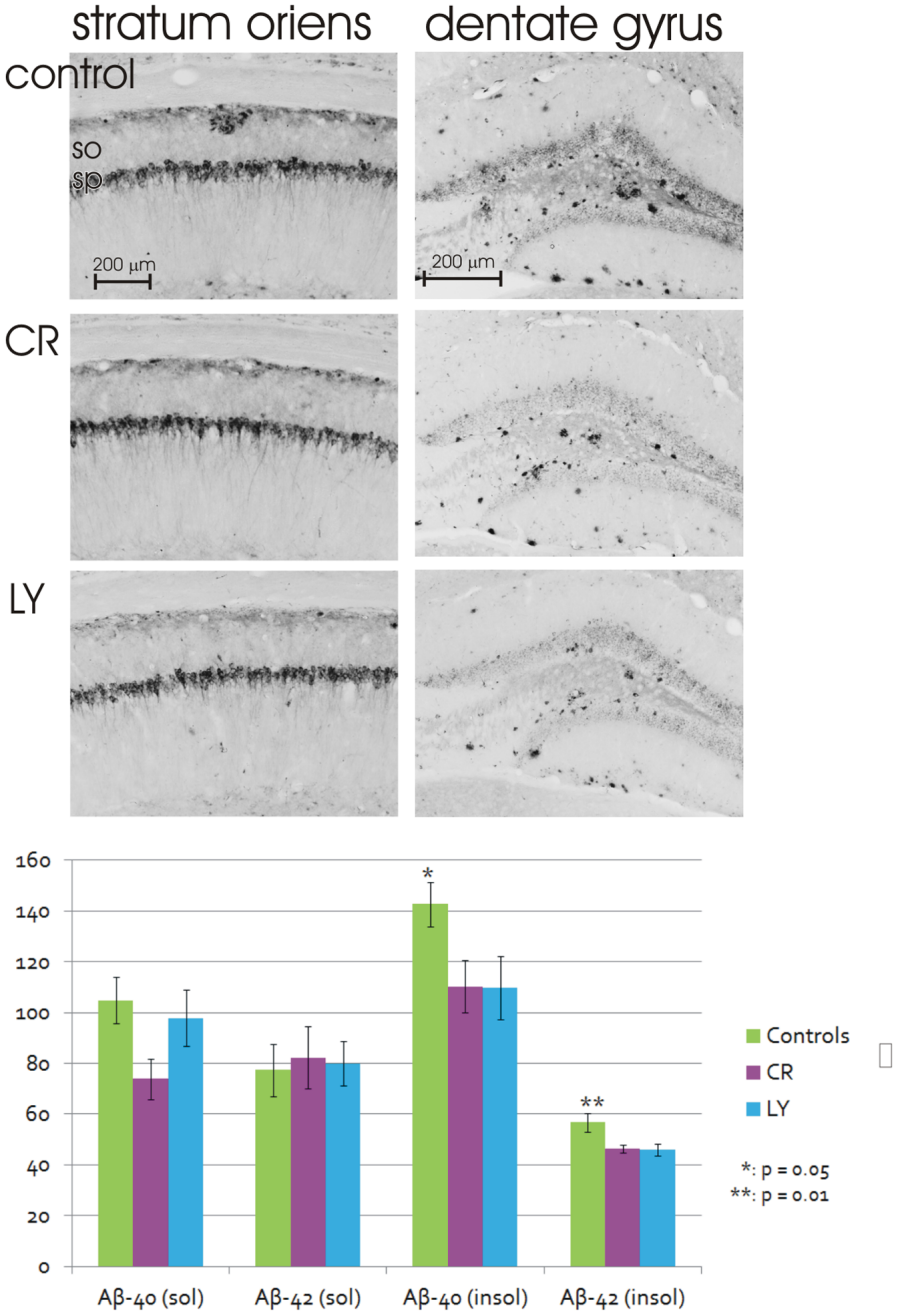
Two series of coronal sections of the dorsal hippocampus stained for Aβ. A non-significant decrease in Aβ staining in stratum oriens and a significant reduction in Aβ staining in the dentate gyrus in both LY and CR mice compared to the control is shown. Lower panel, Aβ levels measured by ELISA. Insoluble Aβ-40 levels are significantly reduced in CR and LY groups compared to the control (*student's t-test p = 0.03 for both comparisons). Insoluble Aβ-42 levels are reduced in CR and LY compared to the control (**Tukey-Kramer HSD p = 0.02 for both comparisons). so - stratum oriens; sp - stratum pyramidale.

AD pathology is characterized by amyloid beta (Aβ) plaques and neuroinflammation. The APPSwDI model of AD starts to develop this pathology at four months of age [20] and was examined after 16 weeks of experimental feeding, i.e., at 6 months of age. Measurement of the Aβ load in the dorsal hippocampus indicated a non-significant reduction in Aβ accumulation in the dorsal hippocampus in area CA1 (stratum oriens) in the CR and LY animals (control, 0.95±0.02; LY, 0.89±0.02 and CR, 0.85±0.02%, respectively; Wilcoxon p = 0.08); however, a significant reduction in Aβ deposition was present in the dentate gyrus in both CR and LY mice (control, 3.95±0.83; LY, 2.05±0.26 and CR, 1.28±0.17%, respectively; Wilcoxon p = 0.04), as measured in immunohistochemical stained material (using the W0-2 antibody; Figure 4). For the ELISA analysis of insoluble Aβ-40 levels, a one-way ANOVA was conducted and was significant (F(2,36) = 3.16, p = 0.05). For comparison of insoluble Aβ-42 levels, the one-way ANOVA was significant (F(2,36) = 5.29, p = 0.01). Both LY and CR groups had significantly lower levels of insoluble Aβ40 and Aβ42 (Figure 4).

**Figure 5:**
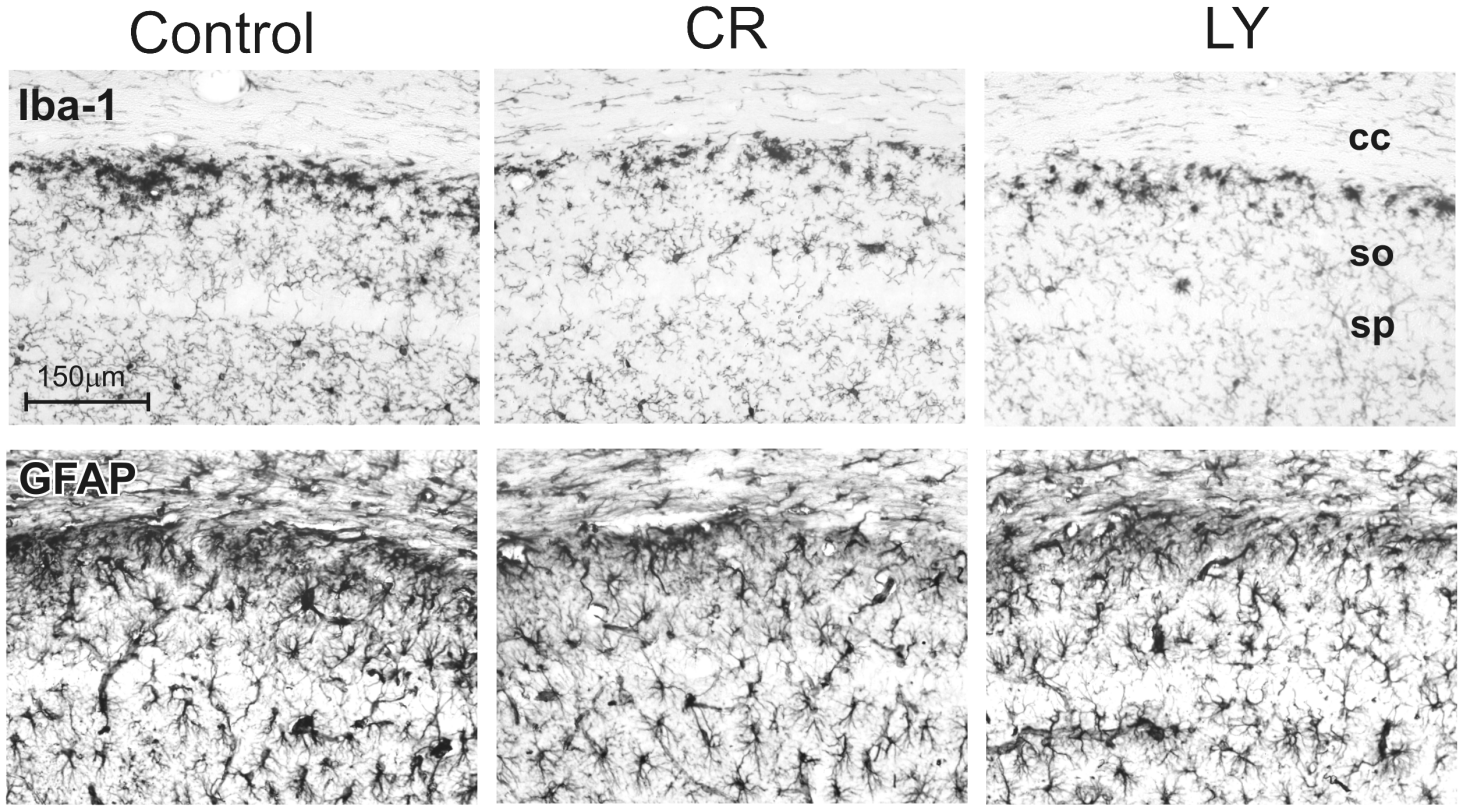
Six high-power photomicrographs of area CA1 of the dorsal hippocampus stained for Iba1 (top row) and GFAP (bottom row), respectively, of the three groups of mice. cc - corpus callosum; so - stratum oriens; sp - stratum pyramidale. Note the significant (Tukey-Kramer HSD p = 0.0042) reduction in Iba1 staining in the CR and LY groups.

**Figure 6:**
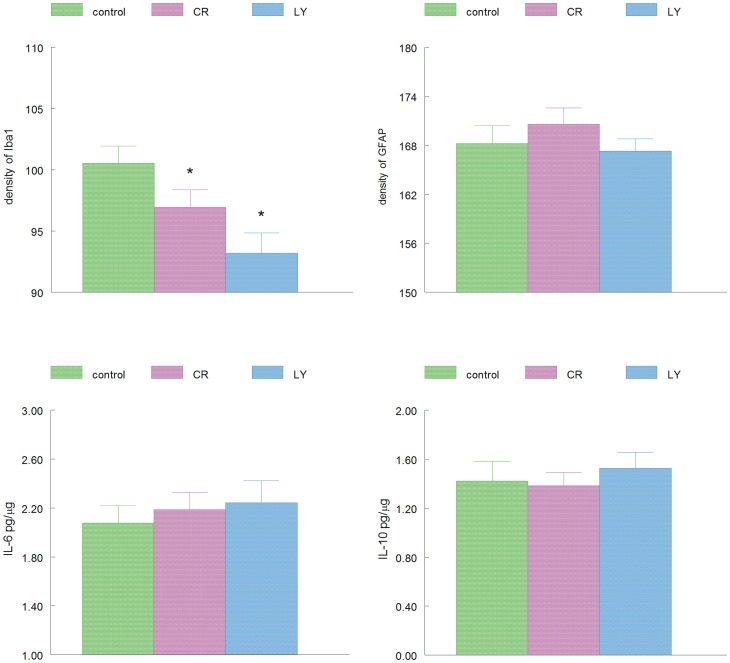
Four bar graphs showing the data from the inflammation measurements, top row, density measurements of Iba1 and GFAP staining in stratum oriens of the hippocampus, respectively (p = 0.0042 for Iba-1). Bottom row, ELISA measurements of IL-6 and IL-10, respectively, levels in the hippocampal formation.

Compared to the control group, LY and CR animals had significantly lower levels of microglial inflammation as indicated by ionized calcium binding adaptor molecule 1 (Iba-1) immunohistochemistry in the dorsal hippocampus (stratum oriens; Tukey-Kramer HSD p = 0.0042, Figures 5 and 6). In contrast, there was no significant change in the GFAP staining density between the groups (p = 0.78, Figures 5 and 6). The ELISA analysis of the cytokine levels of IL6 and IL10 did not reveal a significant difference between the three groups of mice (Figure 6).

## Discussion

These findings demonstrate that treatment with a hunger-inducing ghrelin agonist is sufficient to reduce AD-related cognitive deficits and pathology in Tg AD model mice, similar to the effect of CR on the development of AD pathology.

The LY-treated mice (and not CR mice) significantly improved their performance in the water maze; however, it should be noted that the control mice were not very impaired in their performance. Thus, the lack of significant changes in the CR group compared to the control group may be in part due to a “floor” effect. Furthermore, the CR groups showed a higher level of variation in learning the water maze task between animals. It is of interest to note that the control mice performed better than other groups of these APPSwDI mice of similar age we have tested (unpublished data). It is possible that the increased handling of the mice, by twice weekly weighing and chocolate pellet feeding, reduced their stress levels and thereby improved their performance.

According to the amyloid cascade hypothesis, the primary pathogenesis in AD arises from the formation and deposition of Aβ in the forebrain, which leads to a cascade of pathology [22]. Whereas it previously has been demonstrated that CR significantly reduces amyloid deposition in Tg AD model mice [23], it is likely that the limited change in Aβ levels (i.e., only in the dentate gyrus and not in area CA1) in our CR animals is related to the relatively low level of CR (i.e., only 20%) used in this study compared to the higher levels of CR used in most studies (i.e., 40%). The ELISA data show, however, that there is a reduction in the amounts of insoluble Aβ in both LY and CR groups.

Plaques in this AD mouse model are typically accompanied by activated glial cells, both astrocytes and microglia [24]. The role of the activated glial cells is unclear. On the one hand, they may protect the brain by removing Aβ. On the other hand, they secrete inflammatory cytokines and generate nitric oxide and can thus damage and kill bystander neurons [25]. The role of activated microglia cells in the uptake of Aβ is disputed, with some evidence suggesting Aβ is cleared by microglia [26], whereas other evidence suggests that microglia do not clear Aβ [27]. Plaques in the control animals in our experiment were surrounded by activated glial cells; however, in both the LY and CR mice, the dense amyloid β deposits were associated with much lower activated microglia. This suggests that both CR and ghrelin in the absence of hunger reduce the inflammatory response to deposited Aβ, thus evoking a smaller inflammatory response from microglial cells [28], [29], similar to what has been reported in other studies.

At the end of the study, the CR animals did not show a significant increase in body weight, while both LY and control animals had a similar increase (about 8 grams) in body weight. Similarly, the QMR fat mass data show that the CR mice had significantly lower fat mass compared to the other two groups. Weight loss is a characteristic finding of patients with Alzheimer's disease (AD)[30]–[32]. It seems that it precedes cognitive impairment by some years, but the underlying causes are not fully understood [33]. Both ghrelin and leptin are involved in energy homeostasis and may be implicated in weight loss observed in these patients [34]. Disruption of the normal compensatory modulation of ghrelin secretion in relation to food intake might contribute to the metabolic changes observed in AD patients [35]. The APPSwDI model does not show any weight loss with the onset of dementia similar to most other Tg AD mouse models. The LY mice do not show a change in fat mass with the treatment, compared to the control mice, indicating that our treatment with the ghrelin agonist did not change metabolism.

Several hypotheses have been proposed for the relationship between caloric intake and AD, many of which focus on the amount of calories as the major player in neurodegeneration. Epidemiological studies of obesity, a known risk factor for AD, support the view that dietary factors may influence the severity of dementia [36], [37]. Excessive consumption of calories, particularly fat, opposes healthy brain aging, though the precise mechanisms remain to be elucidated. Evidence from experimental studies in Tg mice [38] suggests that increased Aβ pathology in a transgenic mouse model occurs in association with cholesterol-enriched diets. Thus, hunger and CR may mitigate the formation of amyloid plaques through pathways involved in cholesterol synthesis. Other hypotheses regarding the effects of CR on cognitive decline have thus far focused on the impact of caloric consumption on signaling pathways related to mitochondrial function and oxidative stress; however, our results highlight the potential importance of the hormetic effect of hunger in the mechanism of CR. Overall, our data imply that CR may attenuate development of AD pathology through a neuroendocrine “hunger” signaling pathway rather than a reduced caloric burden.

Several authors have previously entertained the notion that neuroendocrine 'hunger' signaling pathways may be involved in mediating the effects of caloric restriction [39], [40]. For example, Minor et al wrote “Hunger is a fundamental response to CR that triggers a multitude of alterations in the neuroendocrine milieu, and CR modulation of the neuropeptide profile may mediate some of the beneficial changes associated with restrictive diets. Signals like ghrelin from the gut and leptin from adipose tissue converge on the arcuate nucleus of the hypothalamus (ARC) where they are translated into an array of neuropeptides, both orexigenic (e.g., neuropeptide Y (NPY) and agouti-related protein (AgRP)) and anorexigenic (e.g., cocaine- and amphetamine-regulated transcript (CART) and pro-opiomelanocortin (POMC)). These neuropeptides exert potent downstream effects on feeding behavior and other physiological processes including metabolism and circadian rhythms. Neuropeptide Y in particular stands out as a prime candidate for sensing and responding to signals of energy homeostasis as NPY expression levels respond to both short-term and long-term fasting conditions.”[40] Similarly, Hambly et al. [39]wrote “When mice are food deprived, they develop a characteristic neuropeptide gene expression profile in the arcuate nucleus (ARC) of the hypothalamus. This profile involves upregulation of so-called orexigenic neuropeptides (notably NPY and AgRP) that stimulate food intake, and downregulation of anorexigenic neuropeptides that inhibit food intake (POMC and CART). These neuropeptide patterns are believed to underpin the drive to eat and are a molecular marker of the phenomenon of ‘hunger’. All of the aforementioned factors are potential neuroendocrine causes, correlates, or representations of hunger and may mediate the effect of CR on longevity.

Alternatively, it is possible that the LY444711 compound acts through other AD-related pathways induced by ghrelin [40]. Future studies will test other hunger-inducing compounds and a LY444711- treated group fed ad libitum to distinguish the effects of hunger from those of ghrelin. In addition, future studies are needed to better understand how “hunger” without reduced consumption of calories might delay the onset of Aβ pathology and cognitive deficits in APP or APP/PS1 mice and possibly block or delay the onset of AD.
